# CXCR4 and Axillary Lymph Nodes: Review of a Potential Biomarker for Breast Cancer Metastasis

**DOI:** 10.4061/2011/420981

**Published:** 2011-08-23

**Authors:** David Hiller, Quyen D. Chu

**Affiliations:** ^1^Division of Surgical Oncology, Department of Surgery, Louisiana State University Health Sciences Center, Shreveport, LA 71130, USA; ^2^, The Feist-Weiller Cancer Center, LSU Health Sciences Center, 1501 Kings Hwy, Shreveport, LA 71130, USA

## Abstract

CXCR4 is a 7-transmembrane G-protein chemokine receptor that allows for migration of hematopoietic cells from the bone marrow to the peripheral lymph nodes. Research has shown CXCR4 to be implicated in the invasion and metastasis of several cancers, including carcinoma of the breast. CXCL12 is the ligand for CXCR4 and is highly expressed in areas common for breast cancer metastasis, including the axillary lymph nodes. Axillary lymph nodes positive for breast carcinoma have been an important component of breast cancer diagnosis, treatment, and subsequent research. The goal of this paper is to analyze the literature that has explained the pathways from CXCR4 expression to breast cancer metastasis of the lymph nodes and the prognostic and/or predictive implications of lymph node metastases in the presence of elevated CXCR4.

## 1. Introduction

Lymph nodes of the axilla have been studied in the context of breast cancer since before Halsted published his study proposing that this lymphatic drainage was a pathway for metastasis and recommending axillary node dissection (AND) [1]. The understanding of breast cancer metastasis has changed greatly over the past century. Sentinel lymph node biopsies (SNB) allowed for detection of smaller micrometastases that have previously gone undetected. Understanding the true benefit for the use of AND with SNB has been a topic of discussion, and a recent trial has suggested that there is no advantage of AND in patients with a negative SNB [2]. In contrast, an earlier report slightly favored disease-free survival and overall survival in AND patients over just SNB (albeit with a limited number of patients) [3]. This comes 10 years after data was published describing a 30-year followup of internal mammary node dissections that did not improve survival of breast cancer patients [4]. The questioning of the therapeutic value of axillary node dissection in certain patient populations has allowed for further exploration of the prognostic and/or predictive value of these organs. A clearer picture of the molecular mechanisms of lymph node metastases is the next step to designing optimal therapeutic options and to create new treatment modalities. 

One such molecular avenue is chemokine receptor CXCR4, a seven transmembrane G protein-coupled receptor that has been implicated in the invasion and metastasis of several cancers, including breast. Over 20 chemokine receptors have been identified and are keys to pathways that include the body's response to allergy, inflammation, and metastasis. Although a great deal of research into CXCR4 began by focusing on its role in HIV entry of CD4+ cells [5, 6], Müller et al. discovered that CXCR4 was integral for the pathway that activates actin polymerization and pseudopodia formation in breast cancer cells [7]. CXC chemokine ligand-12 (CXCL12), also known as stromal-derived factor-1 (SDF-1), is the ligand for CXCR4. Müller's lab noted that CXCL12 was found in high concentrations at sites that were common for metastasis, such as brain and bone. For this paper, we will only use the term CXCL12.

A litany of studies have emerged in the last five years examining CXCR4 and axillary lymph nodes, covering multiple approaches of how CXCR4 in the setting of axillary lymph nodes can impact our understanding of breast cancer. This review aims to coalesce several papers across the spectrum of this research to tie together molecular pathways and illustrate emerging trends to help direct future research into CXCR4 as a prognostic and/or predictive marker for breast cancer.

## 2. Biology of CXCR4 and Lymph Node Metastasis

CXCR4 was initially discovered as HUMSTR [8] and LESTR [9] and later renamed according to proper nomenclature for chemokine receptors. The action of CXCR4 leads to intracellular signaling cascades that are involved with trafficking, migration, and proliferation. One major site of involvement is in hematopoiesis, due to its expression on CD34+ cells in bone marrow. Evidence points to its role in maintaining hematopoietic progenitor cells [10]. It is also a factor in immune system cells, including monocytes, dendritic cells, NK cells, and naïve T cells. The proposed role of CXCR4 is to help the immune system migrate to sites of injury, but Müller et al. have shown it to be actively involved in metastasis at sites expressing its ligand, CXCL12, playing an important role in the tumor microenvironment [7]. 

The ligand CXCL12 has been further characterized by Crump et al. to understand how it binds to CXCR4 [11]. Their *in vitro* studies revealed CXCL12 to have two binding sites for CXCR4. The RFFESH loop on CXCL12 initially binds with an N-terminal segment of CXCR4. This allows for access to a second receptor site of CXCR4 for the N-terminal region of CXCL12 to bind, altering the conformation of the CXCR4 transmembrane helices and activating the G-protein signal pathway. That signal is able to affect multiple targets, including ERK1/2, MAPK, JNK, and AKT paths, with the end result being events such as chemotaxis, pseudopodia formation, and actin polymeriazation [12–14].

In addition to its role within the immune and hematopoietic systems, CXCR4 has also been implicated as a component in angiogenesis [15, 16]. A well-known component of angiogenesis in tumor cells is vascular endothelial growth factor (VEGF), which has also shown to be prognostic in colon and gastric cancer [17, 18]. CXCR4 has been connected with VEGF, as increased stimulation of CXCR4 leads to increased secretion of VEGF and ultimately angiogenesis and metastasis [19–21]. To achieve metastatic potential, the tumor cells must migrate away from the primary site. Tumor cells must break through the protective extracellular matrix (EM) in order to reach the lymph or blood vessels. Matrix metalloproteinases (MMPs) are essential for this component of invasion, as they cause degradation of EM. One MMP specifically, MMP-9, has been linked to VEGF [22]. 

In addition to CXCR4/CXCL12, VEGF, and MMPs, HIF-1*α* has also shown to be a component of this pathway. HIF-1*α* is a dimeric transcription factor that increases in deoxygenated environments. Hypoxic conditions are known to promote angiogenesis and have been connected to increased HIF-1*α* and VEGF [23]. Additionally, CXCR4 has proven to be increased in hypoxic conditions [24]. The connection between hypoxia, CXCR4, and invasive cancer has been outlined by Sun et al. in a series of experiments that illustrated the connection and molecular pathway between hypoxia in chondrosarcoma, CXCR4 expression, and matrix metalloproteinases. Hypoxic conditions that produced higher levels of HIF-1*α* were observed to subsequently increase CXCR4 expression through the binding of HIF-1*α* to the promoter region of CXCR4. Subsequent CXCR4/CXCL12 signaling via the ERK pathway increased MMP expression and activity [25]. While this pathway was worked out on cell lines for chondrosarcoma, HIF-1*α* increases in hypoxic conditions have also been shown to upregulate CXCR4 in carcinoma of the breast [26]. 

Another component that has been linked to CXCR4 upregulation is nitric oxide (NO). While already known to induce VEGF [27], Nakamura et al. have been able to show that NO also induces the lymphangiogenic factor VEGF-C [28]. In more recent work from their laboratory, MDA-MB-231 cell lines incubated with NO revealed an increased cytoplasmic CXCR4 staining. Cytoplasmic CXCR4 significantly correlated with nitrotyrosine levels, lymph node metastasis, and distant metastasis [29].

The location of CXCR4 staining is a common feature in several of the papers to be described in this review. The CXCR4 receptor resides on the membranes of cells, but IHC has helped reveal patterns between cytosolic and nuclear expression of CXCR4. Tarasova et al. [30] observed endocytosis of membranous CXCR4 receptors in the presence of CXCL12. Salvucci et al. [31] noted that cytoplasmic CXCR4 was seen more often in ductal carcinoma in situ (DCIS) patients when compared to nuclear staining. That group speculated that cytoplasmic CXCR4 staining could be indicative of “active CXCR4 functioning,” as if the cancer cells are ready to leave the primary tumor. While nuclear staining of CXCR4 has been tested and observed in many studies, the reasons behind its difference in expression from cytoplasmic CXCR4 has not yet been uncovered.

**Figure 1:**
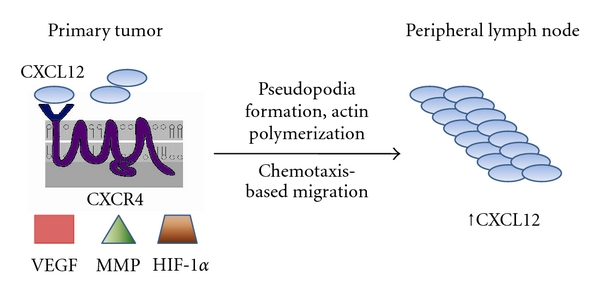
Depiction of a primary tumor overexpressing CXCR4 and the basic factors associated with its migration to peripheral lymph node sites. Increased concentrations of CXCL12 have been noted at lymph node metastasis sites when compared to the primary tumor.

A viable pathway illustrating the role of CXCR4 in breast carcinoma migration from the primary site does not explain how these cells are guided to axillary lymph nodes. Studies have shown CXCL12 to be expressed on the luminal surface of high endothelial venules (HEVs) in peripheral lymph nodes [32]. HEVs are postcapillary venules that enable circulating lymphocytes to enter lymph nodes. CXCL12 is a factor in hematopoietic precursors moving from the bone marrow into the circulation, and ultimately to peripheral tissues [10]. Blades et al. designed a study that showed that CXCL12-induced migration mediated by CXCR4 controlled the migration of human peripheral blood lymphocytes into lymph nodes previously transplanted into SCID mice [33]. Liu et al. performed a series of experiments exhibiting CXCL12 concentrations to be significantly higher in lymph node metastases compared to their primary breast cancer tumor [34]. The location-based chemotaxic ability of CXCL12, combined with its affect on CXCR4-expressed cells, creates a microenvironment for tumor migration (Figure 1).

## 3. CXCR4 Levels and Association to Lymph Node Status

With knowledge of the molecular connections between CXCR4, VEGF, and MMPs, Hao et al. asked if there was a any association between these three components of metastasis and lymph node status in breast cancer patients [35]. The location of CXCR4 staining was studied in breast cancer tissue samples, benign tissue samples that were adjacent to tumor tissue, and atypical hyperplasia samples. CXCR4 was found in both the cytoplasm and the nucleus of mammary cells. Benign tissue adjacent to tumor lesions exhibited weak cytoplasmic CXCR4 staining. The malignant samples had significantly higher rates of CXCR4, VEGF, and MMP-9 compared to the benign and nontumor (hyperplastic) samples (*P* < 0.01). Looking at clinicopathologic factors, statistical significance was found upon analyzing either CXCR4 or VEGF with the tumor's TNM stage. In addition, all three markers had significant association with advanced histologic grade.

When analyzing the primary tumors, increased expression levels of CXCR4, VEGF, and MMP-9 associated with the presence of lymph node positive breast cancer (*P* < 0.001). Results showed a lymph node metastasis rate of 79% with tumors expressing high levels of both CXCR4 and VEGF compared with a 45% rate when only one of these factors is high (*P* = 0.007). In contrast, a 6% rate was observed (*P* < 0.001) when neither factor was highly expressed. Any combination of two of the three markers, each highly expressed, had a significant increase in lymph node metastases. Finally, this study showed that high CXCR4 expression was positively associated with increased VEGF and MMP-9 expression (Table 1). 

Kang et al. have shown that high CXCL12 expression in breast cancer tissue is linked to nodal and distant spread of breast cancer cells, as well as a link to overall survival [36]. To follow up this study, this group focused on CXCR4's relation to metastasis and survival [40], as opposed to its ligand. Using immunohistochemistry, CXCR4 was detected in both breast cancer cell lines as well as normal mammary tissue. It was shown that node-positive tumors had a significantly increased expression of CXCR4 when compared to node-negative tumors. CXCR4 expression was higher in the metastatic cohort compared to the nonmetastatic group, but significance was not achieved when looking at a relationship between elevated CXCR4 and presence of distant metastases or overall survival (Table 1). 

Parker et al. evaluated a cohort of 185 node-positive breast cancer patients and found that CXCR4 overexpression level in primary tumors independently predicted a poor outcome for these patients [37]. The 5-year overall survival for patients with low and high CXCR4 overexpression was 69% and 57%, respectively, (*P* = 0.02, Table 1). These results suggest that even within this high risk group (i.e., node positive patients), CXCR4 can be employed to identify those patients who have a more aggressive disease course and therefore can be targeted for more intensive and/or novel therapy.

Not all studies have successfully concluded CXCR4 to definitely predict outcome. Cabioglu et al. studied CXCR4 as a predictive marker for lymph node metastasis along with CCR7 [38], another chemokine receptor that has been shown to be expressed in breast cancer cells [7]. The group attempted to reduce confounding effects that can accompany T2-4 lesions by limiting this study to only T1 lesions. Differences in CXCR4 and CCR7 staining location were tested, with node-positive tumors showing higher cytoplasmic CCR7 and HER2 staining than node-negative tumors. There was an increased rate of CXCR4 in node-positive patients (11.2% versus 5.1%), but this difference was not significant (Table 1). The authors theorized that CCR7 is associated with lymph node metastasis, while CXCR4 expression aids in the reliability of CCR7 as a biomarker. When Liu et al. investigated the relationship between CXCR4 and CCR7, they found in their data set that CXCR4 and CCR7 each significantly associated with lymph node metastasis. A lower overall survival was noted via the Kaplan-Meier survival analysis of both CXCR4 and CCR 7 overexpression [34].

 The difference between nuclear and cytoplasmic CXCR4 staining has emerged as an important factor in CXCR4's prognostic/predictive ability [41]. Su et al. examined the expression location of CXCR4 in breast cancer cells and tested for associations in marker staining and metastasis [39]. The study was designed to compare 3 groups: (1) patients with sentinel and nonsentinel lymph node metastasis, (2) patients with only sentinel lymph node metastasis, and (3) patients with neither sentinel or nonsentinel metastasis. Combining groups 2 and 3 together (they chose for sole sentinel node positivity to be equivalent to no metastasis) revealed that high cytoplasmic CXCR4 expression was associated with axillary lymph node status (*P* = 0.0325, Table 1). Their data did not show any other correlations with cytoplasmic or nuclear CXCR4 staining and any other clinicopathological factor. Essentially, these results show that elevated cytoplasmic CXCR4 are indicative of spread beyond the sentinel lymph node.

## 4. Autocrine versus Paracrine CXCL12

The knowledge that stromal cells express CXCL12 opens the theory that location, along with concentration, can be important in predicting outcome. Kang et al. found that when adding CXCL12 to the cell line MDA-MB-231, there was increased migration and invasion ability. That study showed an inverse relationship between CXCL12 expression levels and disease-free and overall survival in breast cancer patients. Increased CXCL12 caused an increased incidence of recurrence and lymph node metastasis [36]. 

Mirisola et al. conducted a series of experiments to help explain the autocrine/paracrine effect of CXCL12 [42]. When analyzing 100 breast cancer samples, IHC studies showed that groups highly expressing CXCL12 tended to be smaller tumors and lymph node negative (*P* = 0.04, *P* = 0.002). Also noted was a significant association between DFS and the expression pattern of CXCL12, specifically expression at the tumor periphery (*P* = 0.002). CXCR4 expression in this data was not significant for DFS or OS. Tumors expressing CXCL12 had a better clinical outcome than those that lost the chemokine. The authors' explanation is that when CXCL12 is produced by the tumor, the autocrine function of CXCL12 renders the tumor insensitive to its effects and cancels any metastatic potential. Tumor growth via the ERK pathways and VEGF are still in play, but metastatic potential is lost. Thus, it can be inferred that tumors not overly expressing CXCL12 show a paracrine effect and maintain metastatic potential.

The idea that CXCL12 can inhibit the effect of CXCR4 was further explored by Shim et al. [26]. First, they were able to produce results that showed breast cancer lymph node metastases were overexpressed at a lower rate than they were at the primary tumor. Next, they moved to explain how increased CXCL12 concentrations can affect CXCR4, a finding previously noted in Liu et al. [34]. After creating an “expression score” to quantify CXCR4 expression in their breast cancer and lymph node specimens, this group was able to show that primary breast cancer specimens exhibited a higher score than lymph node metastases (*P* < 0.001). Two immunostaining patterns were noticed. 58% of primary tumor samples had membranous staining of CXCR4 predominate, versus 80% of lymph node metastases having cytoplasmic staining predominate. In the reviewed papers Hao et al. [35], Su et al. [39], Yasuoka et al. [29], and Liu et al. [34] each of these observed cytoplasmic staining to associate with lymph node metastasis. 

This group studied CXCL12 mRNA levels of 10 primary breast cancer tumors along with their matching lymph node metastases and observed CXCL12 levels to be higher in lymph node tumors than the primary breast tumor (*P* < 0.001). For a control, the MDA-MB-231 cell line was examined. CXCR4 staining was observed to be predominantly membranous in this line. However, after incubation with CXCL12 for 30 minutes, CXCR4 expression was found in the cytoplasm. Furthering this experiment, MDA-MB-231 cells were exposed to 100 and 200 ng/mL concentrations of CXCL12 for 48 hours. CXCR4 was then measured by Western blot, and expression as found to be significantly decreased. This group then tested chloroquine, known to inhibit proteolysis of lysosomes, with the MDA-MB-231 cell line and found CXCR4 expression returned while still in the presence of high concentrations of CXCL12. This suggests that high CXCL12 concentrations cause cellular degradation of CXCR4 receptors.

## 5. Alternate Research Pathways and Questions

It has been widely accepted that CXCL12 is the exclusive ligand of CXCR4. Burns et al. have altered this belief with a study documenting the ability of CXCL12 to bind to CXCR7 (RDC1, CCX CJR2), a novel receptor that was first characterized as a chemokine receptor in this paper [43]. They were able to show that CXCL12 binds to CXCR7 by transfecting a cell line lacking CXCR4 and CXCR7 with the RDC1 gene, resulting in high-affinity CXCL12 binding even in the presence of the CXCR4 inhibitor, AMD3100. In the following experiments, Burns et al. introduced CXCR7 into breast cancer cell line MDA MB435s and documented an increase in cell growth and increased adhesion to human umbilical vein endothelial cells. This lab's continued investigation of CXCR7 has since shown that it promotes breast and lung cancer in murine models [44]. Additionally, CXCR7 was undetectable or at low levels in normal human breast tissue from mammoplasties, but was clearly detected in over 30% of human breast cancer specimens. It was also detectable in 97% of blood vessel specimens from human breast cancer, versus being “undetectable or nearly undetectable” in normal blood vessels from normal breast tissue.

Searching for new biomarkers is more complex than locating factors with changed expressions from their benign baseline, according to Ransohoff and Gourlay [45]. They state that several forms of bias might account for the fact that while many targets have been identified as biomarkers, very few have had “clinical value.” This bias possibly occurs before a specimen arrives to a laboratory in the form of collection and storage. One example cited details a group investigating prostate cancer that questioned whether differences in storage time between the cancer and noncancer specimen groups affected the end results [46]. Ransohoff and Gourlay's assessment of bias concludes that subject selection is of utmost importance—“inequality of specimen groups” is a major source of bias in experiments where the outcomes are observations, not laboratory results. They believe that improving the attention given to specimens, both in selection and management once acquired, might improve the quality of results in biomarker identification research.

## 6. Conclusion

CXCR4 is an important factor in breast cancer metastasis. The molecular pathway for its action, along with associated factors, is continuing to be broken down piece by piece. A myriad of components appear to play important roles in the overexpression of CXCR4, from NO and VEGF to MMPs and HIF-1*α*. Differences have been noted in CXCR4 between primary tumors and lymph node metastases, specifically in the amount of overexpression: CXCR4 has been found to be more highly overexpressed at the primary tumor than at lymph node metastases. Repeated in many papers is the fact that cytoplasmic CXCR4 staining is noted to associate with lymph node metastasis while nuclear CXCR4 staining has not had significant results. Studies into the responsibilities of CXCR4's ligand, CXCL12, have revealed increased concentration of the ligand at distant sites, specifically the lymph nodes. Another important issue with CXCL12 is the idea that tumors that produce high amounts of the chemokine effectively downregulate CXCR4 receptors, while tumors without high CXCL12 expression maintain a prometastatic ability. The role of CXCR7 further clouds the picture when attempting to understand what is more important to target while grasping CXCL12's effect on both CXCR7 and CXCR4 in the same tumor microenvironment. 

CXCR4 has been identified as a receptor for CXCL12, changes in expression location patterns of CXCR4 have been described, and positive associations with disease outcome have been derived. New discoveries concerning CXCR7, paracrine/autocrine CXCL12 effects, and more careful planning in discovery and examination of biomarkers will help shape the future directions of this research. Possibilities exist for collaboration and shared information; the results of which could alter the current understanding and treatment of breast cancer, both local and systemic.

## Figures and Tables

**Table 1:** CXCR4 and lymph node metastasis in recent studies.

Study	Findings	*P* value
Hao et al. [35]	↑CXCR4 with ↑TNM stage	<0.037
	↑CXCR4 with +lymph node mets	<0.001
	↑CXCR4/VEGF w/+LN mets	0.007
	↑CXCR4/MMP-9 w/+LN mets	<0.001

Kang et al. [36]	↑CXCR4 in +LN tumors over −LN tumors	0.03
	↑CXCR w/OS or distant mets-not observed in this data	—

Parker et al. [37]	↑CXCR4 w/+LN had worse 5 yr OS	0.02

Cabioglu et al. [38]	↑CXCR4 w/+LN	0.113

Su et al. [39]	↑CXCR4 (cytoplasmic) with +LN mets	0.0325

CXCR4: CXC chemokine receptor 4, LN mets: lymph node metastases, MMP-9: matrix metalloproteinase-9, VEGF: vascular endothelial growth factor.
